# Impact of the 2018 Japan floods on asthma prescriptions: A longitudinal analysis

**DOI:** 10.1016/j.jacig.2026.100792

**Published:** 2026-10-05

**Authors:** Hanae Konishi, Shuhei Yoshida, Hiroshi Iwamoto, Yasushi Horimasu, Shu Utsumi, Shinichiro Ohshimo, Noboru Hattori, Masatoshi Matsumoto

**Affiliations:** a Department of Molecular and Internal Medicine, Graduate School of Biomedical and Health Sciences, Hiroshima University, Hiroshima, Japan; b Department of Community-Based Medical Systems, Graduate School of Biomedical and Health Sciences, Hiroshima University, Hiroshima, Japan; c Department of General Internal Medicine, Hiroshima University Hospital, Hiroshima, Japan; d Department of Emergency and Critical Care Medicine, Graduate School of Biomedical and Health Sciences, Hiroshima University, Hiroshima, Japan

**Keywords:** Climate change, epidemiology, exacerbation, natural disaster

## Abstract

**Background:**

The impact of flood disasters on adult asthma is poorly understood.

**Objective:**

This study aimed to investigate the impact of the 2018 Japan floods on new prescriptions for asthma controller medications and treatments for asthma exacerbations in adults.

**Methods:**

This retrospective longitudinal analysis used data from the National Database of Health Insurance Claims and Specific Health Checkups for residents of regions severely affected by the 2018 Japan floods, covering 1 year before and 1 year after the disaster. New asthma controller prescriptions were compared between officially certified disaster victims and nonvictims with no controller use in the preceding year. Among prior controller users, prescriptions for short-acting *β*_*2*_-agonists and oral corticosteroids were compared between victims and nonvictims.

**Results:**

Among 5,000,129 participants, 31,235 were disaster victims. Victims had a higher incidence of new asthma controller prescriptions than nonvictims (adjusted hazard ratio, 1.47; 95% CI, 1.38-1.57). This increase was observed across all age groups and was greater in younger and middle-aged adults. A difference-in-differences analysis showed increased short-acting *β*_*2*_-agonist prescriptions at 3, 5, 7, and 11 months after the disaster and increased oral corticosteroid prescriptions at 5 months among victims.

**Conclusions:**

Victims of the 2018 Japan floods had increased prescriptions for new-onset and exacerbated asthma, suggesting an association between flood disasters and increased adult asthma-related health care needs.

Climate change has led to an increase in extreme rainfall events and flood disasters.^1^, ^2^ The increasing frequency of heavy rainfall and flood disasters is likely to contribute to a higher incidence of respiratory diseases, highlighting the importance of appropriate countermeasures.^3^ Flood victims may be at elevated risk of asthma onset and exacerbation because of exposure to allergens, pollutants, pathogens, and psychological stress.^4^, ^5^ A household survey of flood-affected residents in Detroit found that among 3,842 households, 2,085 with flooded homes reported an increase in asthma cases (odds ratio, 1.42; 95% CI, 1.22-1.64).^6^

An analysis of Tropical Storm Imelda in Texas reported increased asthma-related emergency department visits among flood victims, particularly in children.^7^ Increased asthma-related emergency visits were also reported 1 month after Hurricane Floyd, compared with the same period the previous year (odds ratio, 1.4; 95% CI, 1.2-1.7).^8^ Recently, our research group demonstrated a significant increase in new pediatric asthma prescriptions following the 2018 Japan floods.^9^ However, whether the impact of flood disasters on adult asthma persists beyond the immediate postdisaster period remains unclear. From June 28, 2018, to July 8, 2018, western Japan experienced severe rainfall, which led to landslides and flooding. This was the largest flood disaster in recorded Japanese history, with 263 fatalities, 484 injuries, and 8 missing persons. Additionally, 6,783 homes were destroyed, and 44,327 were damaged.^10^ Hiroshima, Okayama, and Ehime Prefectures were the hardest-hit areas, accounting for approximately 90% of total fatalities.

This study aimed to investigate the impact of flood disasters on new prescriptions for asthma controller medications and treatments for asthma exacerbations among adults. To this end, we analyzed large-scale prescription data from the most severely affected areas and compared disaster victims with nonvictims over a 1-year period following the 2018 Japan floods.

## Methods

This retrospective cohort analysis used data from the National Database of Health Insurance Claims and Specific Health Checkups (NDB), managed by the Japanese government. Under Japan’s universal health insurance system, all citizens, except <2% receiving public assistance, are enrolled in public health insurance, ensuring that the NDB contains detailed records of all prescriptions and insurance claims nationwide. This study included individuals aged ≥20 years who visited medical institutions in Hiroshima, Okayama, and Ehime Prefectures during the observation period (Fig 1). We extracted demographic data (age and sex) and prescription records from the NDB over 2 years (July 2017 to June 2019), covering 1 year before and 1 year after the disaster.

**Fig 1:**
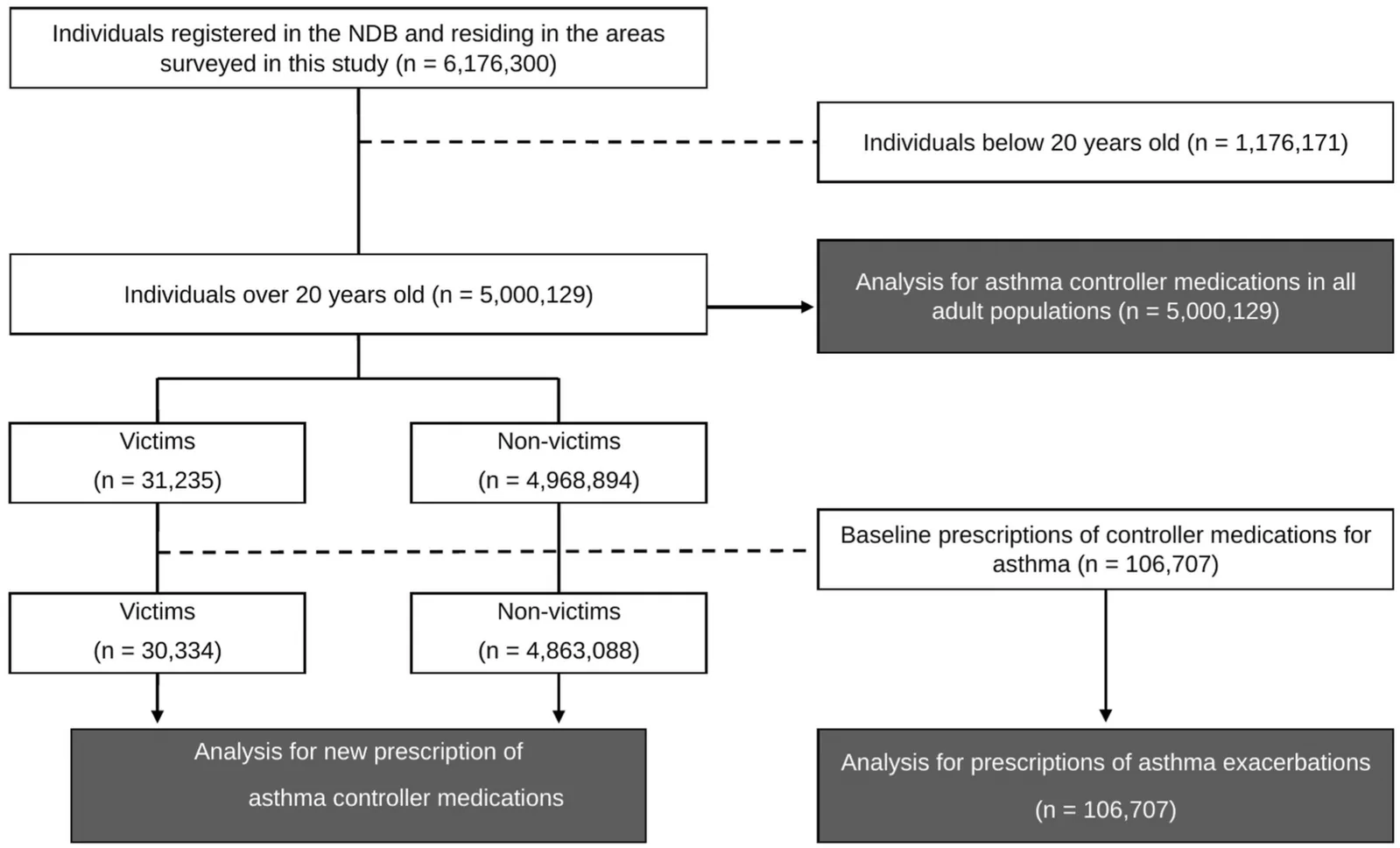
Study flow.

Because the NDB data were anonymized, the requirement for informed consent was waived. This study was approved by the Hiroshima University Epidemiological Research Ethics Committee (approval number E-1688), and data usage was authorized by the Ministry of Health, Labour and Welfare (approval number 1223-2).

### Definition of disaster victims

Individuals officially certified as disaster victims following the 2018 Japan floods were designated as disaster victims in the NDB. Disaster victim status was determined according to the certification criteria established by local authorities. Disaster victims were defined as follows:

1. A family member who financially supported the disaster victim was killed, injured, missing, unemployed, out of work, or without income.
2. A residential house was completely or partially destroyed, flooded, or burned down.

### Outcomes

The study first assessed new prescriptions for asthma controller medications, including inhaled corticosteroids and inhaled corticosteroids/long-acting *β*_*2*_-agonists, within 1 year after the flood disaster among individuals without a history of prescriptions in the year before the disaster (Fig 1; see Table E1 in this article’s Online Repository at www.jaci-global.org). A sensitivity analysis was conducted to compare prescription trends for asthma controller medications between victims and nonvictims across all adult populations, providing a comprehensive assessment of the disaster’s impact (Fig 1).

Second, for patients with asthma who had been prescribed asthma controller medications in the year before the disaster, prescriptions for short-acting *β*_*2*_-agonists (SABAs) and oral corticosteroids (OCS) were analyzed separately and compared between victims and nonvictims to assess trends in exacerbation treatment (Fig 1 and Table E1).

### Statistical analysis

Categorical variables are presented as numbers and percentages. Age was categorized as young adulthood (20-39 years), middle age (40-64 years), and older age (≥65 years). A logistic regression analysis was performed to examine the association between the presence or absence of asthma controller medication prescriptions in the year before the disaster (the dependent variable), adjusting for age and sex. Subgroup analyses of new asthma controller medication prescriptions were conducted by age and sex.

For individuals without a prior prescription for asthma controller medications in the year preceding the disaster, the cumulative incidence of new prescriptions was estimated using the Kaplan-Meier method. Differences between disaster victims and nonvictims were assessed using a log-rank test. Furthermore, a Cox proportional hazards model was used to compare new prescriptions for asthma controller medications between disaster victims and nonvictims, adjusting for age and sex.

For the sensitivity analysis, a difference-in-differences (DID) analysis was conducted to assess trends in the prescribing of asthma controller medications in the overall adult population. DID analysis is a quasi-experimental method that compares changes in prescription incidence between disaster victims and nonvictims, theoretically controlling for potential confounding factors. The analysis assumes parallel trends (ie, in the absence of exposure, differences in prescription rates between the 2 groups would remain constant) and common shocks (ie, any external event will affect both groups equally). In the DID analysis, we compared prescription incidence in May 2018 (2 months before the disaster) with each subsequent month from June 2018 onward. The estimated change in prescription rates among disaster victims relative to nonvictims was calculated using a multivariable logistic regression model with a time interaction term, and the results were expressed as the ratio of odds ratios (ROR). Adjusted RORs with 95% CIs were reported for each month after June 2018, with May 2018 as the reference.

The DID analysis was conducted using a linear probability model with an interaction term for disaster status (victims vs nonvictims). Robust SEs (cluster subcommands) were used to account for repeated measurements within individuals. Finally, a separate DID analysis was conducted to assess the risk of asthma exacerbation among patients prescribed asthma controller medications in the year preceding the disaster.

All statistical analyses were conducted using Stata/MP Parallel Edition, version 16.1 (Stata Corp LLC, College Station, Tex). Statistical significance was set at a 2-tailed *P* value < .05.

## Results

### Baseline characteristics

The study population comprised 5,000,129 individuals aged ≥20 years, selected from 6,176,300 patients recorded in the NDB who had either outpatient visits or hospitalizations at medical institutions in Hiroshima, Okayama, and Ehime Prefectures (Fig 1). Of these participants, 31,235 were disaster victims, and 4,968,894 were nonvictims. Table I presents baseline characteristics of disaster victims and nonvictims. More than 50% of the disaster victims were aged ≥65 years, a higher proportion than that observed among nonvictims. Patient characteristics of those receiving asthma controller medications in the year preceding the disaster are provided in Table E2 (in the Online Repository available at www.jaci-global.org). A logistic regression analysis showed no significant difference in asthma controller medication prescriptions between disaster victims and nonvictims in the year before the disaster (adjusted odds ratio, 1.05; 95% CI, 0.998-1.112; *P* = .082) (see Table E3 in this article’s Online Repository at www.jaci-global.org).

**Table I:** Characteristics of the study cohort

Characteristic	Victims (n = 31,235) n (%)	Nonvictims (n = 4,968,894) n (%)
Age category (y)		
20-39	4,654 (15)	1,302,881 (26)
40-64	10,185 (33)	1,870,036 (38)
≥65	16,396 (52)	1,795,977 (36)
Sex		
Male	13,583 (43)	2,304,674 (46)
Female	17,652 (57)	2,664,220 (54)

### Increase in new prescriptions for asthma controller medications among disaster victims

The characteristics of individuals with new prescriptions for asthma controller medications after the disaster are provided in Table E4 (in the Online Repository available at www.jaci-global.org). The cumulative incidence of new prescriptions for asthma controller medications was higher among disaster victims than among nonvictims (*P* < .01) (Fig 2). A multivariable Cox proportional hazards analysis revealed a significant increase in new prescriptions for asthma controller medications among disaster victims compared with nonvictims (adjusted hazard ratio, 1.47; 95% CI, 1.38-1.57) (Table II). Subgroup analyses across all age groups (young adults, middle-aged adults, and older adults) showed that the incidence of new prescriptions was significantly higher among disaster victims than among nonvictims (Fig 3, *A-C*). The multivariable Cox proportional hazards model indicated a higher likelihood of receiving a new prescription for asthma controller medications among young and middle-aged adults than among older adults (Table II and see Table E5 in this article’s Online Repository at www.jaci-global.org). In the sex-stratified analysis, both men and women showed an increased risk of receiving new prescriptions for asthma controller medications after the disaster (Fig 3, *D* and *E*), with a significantly higher risk in women than in men (Table II and Table E5).

**Fig 2:**
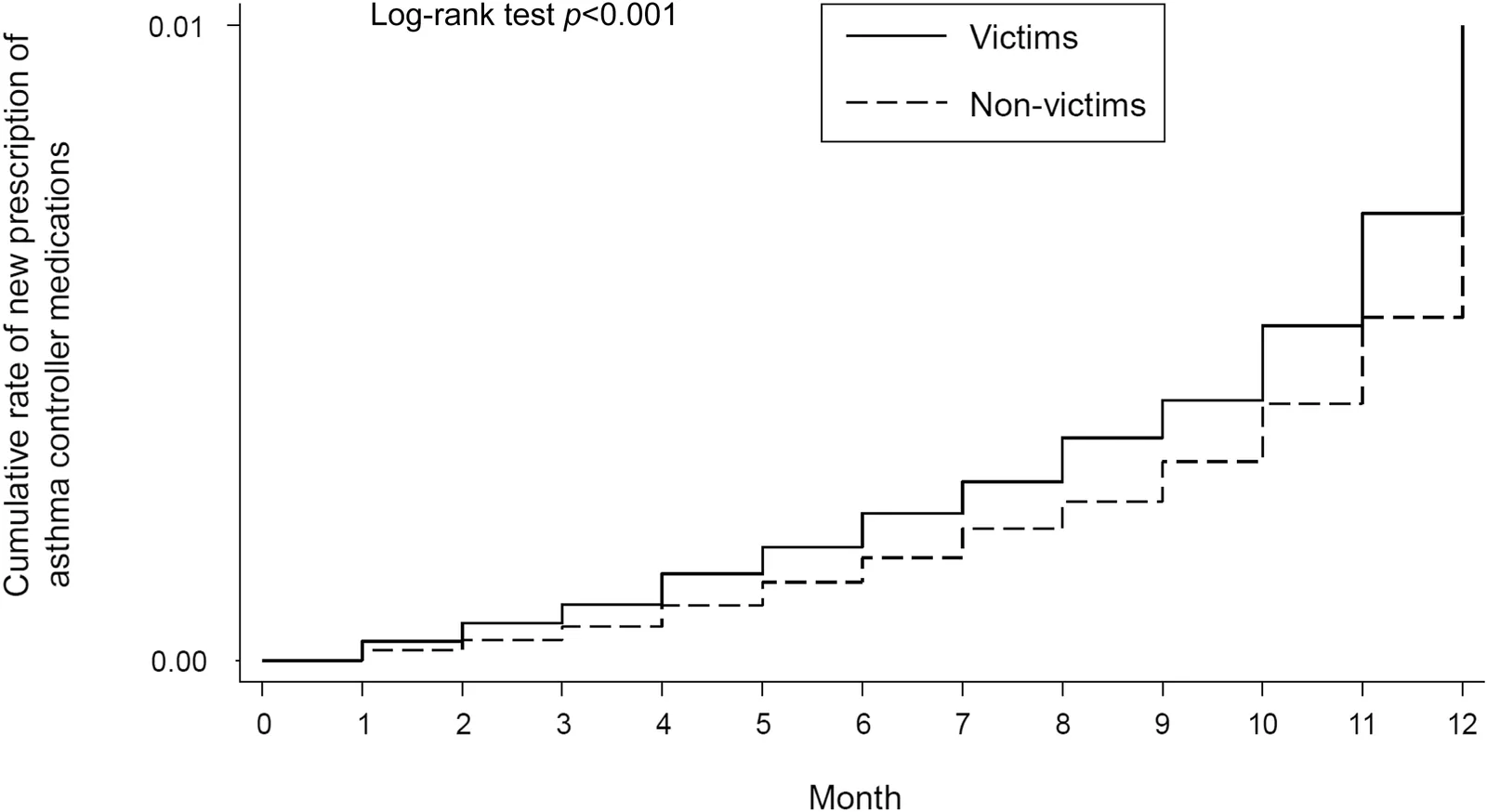
Cumulative incidence of new prescriptions for asthma controller medications. Kaplan-Meier curve showing a significant increase in new prescriptions for asthma controller medications among disaster victims compared with nonvictims during the 1-year period after the floods. Month 0 corresponds to July 2018, when the floods occurred.

**Table II:** Multivariate Cox proportional hazards analysis for new prescriptions of asthma controller medications after the disaster

Variable	Hazard ratio (95% CI)	*P* value
Nonvictims	Reference	
Victims	1.47 (1.38-1.57)	<.001
Age (y)		
20-39	1.84 (1.81-1.87)	<.001
40-64	1.59 (1.57-1.62)	<.001
≥65	Reference	
Sex		
Male	Reference	
Female	1.48 (1.46-1.50)	<.001

**Fig 3:**
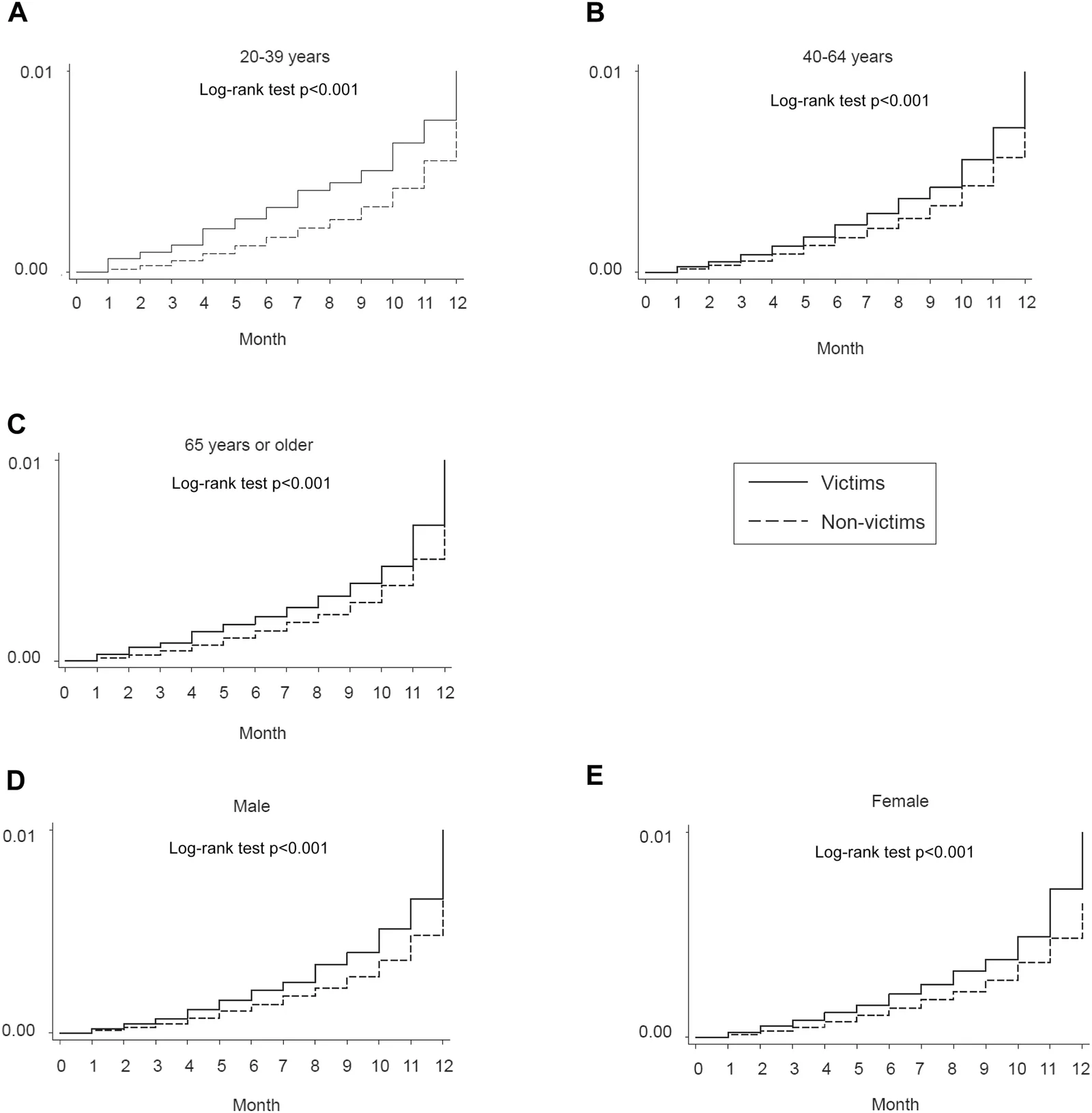
Subgroup analysis of new prescriptions for asthma controller medications by age and sex. Individuals aged **(A)** 20 to 39 years, **(B)** 40 to 64 years, and **(C)** ≥65 years; and **(D)** males and **(E)** females. Month 0 corresponds to July 2018, when the floods occurred.

### Increased risk of asthma controller medication prescriptions among disaster victims

To assess the overall impact of disaster on asthma controller medication prescriptions, we conducted a sensitivity analysis in the overall adult population. The DID analysis revealed that the adjusted ROR for prescriptions among disaster victims relative to nonvictims increased immediately after the disaster (adjusted ROR, 1.22; 95% CI, 1.1-1.34) and remained significantly elevated even 1 year later (adjusted ROR, 1.39; 95% CI, 1.26-1.53) (see Fig E1 in this article’s Online Repository at www.jaci-global.org).

### Increased prescriptions for asthma exacerbations among disaster victims

We analyzed asthma exacerbation prescriptions for 1,288 disaster victims and 191,394 nonvictims who had been prescribed asthma controller medications in the year before the disaster. The DID analysis showed a significant increase in the ROR for SABA prescriptions among disaster victims compared with nonvictims at 3, 5, 7, and 11 months after the disaster (Fig 4, *A*). The ROR for OCS prescriptions was significantly higher in disaster victims than in nonvictims at 5 months after the disaster (adjusted ROR, 1.35; 95% CI, 1.07-1.76; *P* = .026) (Fig 4, *B*).

**Fig 4:**
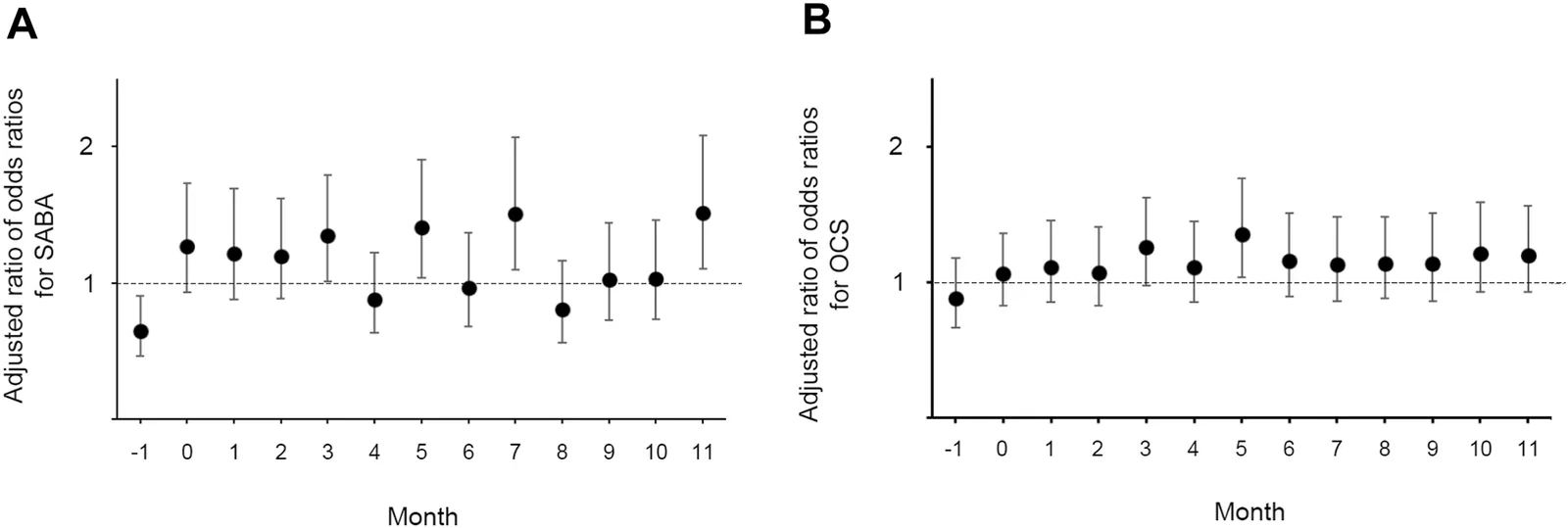
Adjusted RORs for prescriptions for asthma exacerbations among victims compared with nonvictims. The prescription rate was compared among individuals who had been prescribed asthma controller medications the year before the disaster and was evaluated using DID analysis (n = 106,707). (A) SABA and (B) OCS. Month 0 corresponds to July 2018, when the floods occurred. RORs were adjusted for age and sex. The *dotted horizontal line* indicates an adjusted ROR of 1.

## Discussion

This study examined prescription patterns for asthma medications among disaster victims in the first year after the 2018 Japan floods, using large-scale health insurance data. Our findings demonstrate that new prescriptions for asthma controller medications were significantly higher among disaster victims than among nonvictims. Additionally, among patients who had been receiving asthma controller medications before the disaster, prescriptions for exacerbation treatments were significantly higher among disaster victims than among nonvictims. Previous studies have reported prolonged exposure to house dust, mold, and air pollutants from flooded buildings, which can persist long after a flood disaster.^11^, ^12^ Furthermore, changes in living conditions, psychological stress, and clean-up activities involving exposure to debris and contaminated air can adversely affect respiratory health.^10^, ^13^ Flooding has also been associated with an increased risk of influenza infections, which may have contributed to a delayed increase in prescriptions for exacerbation treatments during the subsequent winter.^14^ These contributing factors may have increased the burden of new-onset asthma and asthma exacerbations during the first year after the disaster. Future disaster response strategies should include measures to mitigate these risks and protect the respiratory health of affected populations.

This study examined the impact of flood disasters on adult asthma across different age groups and found a greater increase in demand for asthma controller medications among young and middle-aged adults than among older adults. A previous report found that allergen-specific IgE levels are higher in young adults than in older populations,^15^ which may increase the risk of asthma onset because of allergen exposure. Additionally, previous reports showed that air pollution increases asthma risk in younger populations.^16^, ^17^ The present study showed that older disaster victims had a significantly higher rate of prescriptions for asthma controller medications than nonvictims, although the magnitude of the risk increase was lower than that observed in younger individuals. Previous studies have reported increased asthma-related hospitalizations among older adults because of air pollution.^18^ Furthermore, environmental factors and psychological stress related to disaster exposure can affect individuals of all ages, potentially exacerbating asthma symptoms.^19^, ^20^ Our findings underscore the importance of monitoring all adult age groups for increased asthma risk in the aftermath of flood disasters.

In the sex-stratified analysis, new prescriptions for asthma controller medications were significantly higher among disaster victims than among nonvictims in both men and women, with a more pronounced increase in women postdisaster. Female predominance in asthma after puberty and greater susceptibility to environmental and psychological stress may partly explain this more pronounced increase in women.^21^, ^22^ Hormonal factors, such as estrogen, which regulates immune responses and stress pathways, may also have contributed to this sex difference.^23^

Our series of nationwide studies consistently showed associations between the 2018 Japan floods and increased prescriptions for pediatric and adult asthma, allergic rhinitis, and chronic obstructive pulmonary disease (COPD).^9^, ^24^, ^25^ Although a common environmental exposure may underlie these observations, susceptible populations varied by disease. In our previous pediatric asthma study,^9^ the increase in new prescriptions was greater among children aged ≥10 years, and boys had a higher risk of receiving new prescriptions than girls. In contrast, the present study found a greater increase in new controller prescriptions among younger and middle-aged adults than among older adults, with a more pronounced increase in women. In our allergic rhinitis study,^24^ prescription rates increased across all age groups and both sexes, whereas the COPD study^25^ demonstrated a significant increase primarily among men, with a delayed increase observed in older adults. These findings suggest that flood disasters affect multiple respiratory diseases through shared environmental and psychosocial mechanisms, whereas the disease magnitude, timing, and susceptible populations vary by disease type, highlighting the clinical relevance and novelty of the present study.

This study had several limitations. First, the database does not include patient-specific diagnostic information; therefore, asthma could not be completely distinguished from COPD. Second, because information on potential confounding factors such as smoking and socioeconomic status was not available, these variables could not be directly accounted for in the analysis. However, we used the DID method to minimize the potential effects of confounding factors. Third, we could not conduct a subgroup analysis of prescriptions for asthma exacerbations because of the limited number of patients who experienced exacerbations during the observation period. Fourth, because the NDB recorded disaster victim status as a single variable, we could not distinguish individuals certified for direct housing damage from those certified for loss of livelihood. Therefore, heterogeneity in the degree of flood exposure could not be evaluated. Fifth, the increase in prescriptions might have been influenced by the exemption from copayments for disaster victims. Certified victims received temporary exemptions from out-of-pocket medical expenses after the floods. However, drug prices in Japan are generally lower than in other countries,^26^ and the maximum copayment rate under Japan’s public health insurance system is only 30%. Notably, the copayment rate varied by age (10%, 20%, and 30% for those aged ≥75 years, 70-74 years, and <70 years, respectively), and the calculated average copayment rate for the disaster-affected group in this study was 22.3%, which is relatively low. Additionally, the price elasticity of demand for outpatient care in Japan is reported to be low (−0.125 to −0.07619).^27^ Therefore, the impact of the copayment exemption on the study results is likely minimal. Sixth, SABA and OCS prescriptions may be influenced by seasonal variation. Although the DID analysis compared disaster victims and nonvictims over the same calendar months, thereby partially accounting for shared seasonal effects, residual seasonal variation cannot be completely excluded. Finally, we could not track individuals who moved out of Hiroshima, Okayama, or Ehime Prefectures during the study period. However, in 2019, approximately 1.6% of the total population relocated outside the study area, indicating a minimal impact on our findings. Moreover, our study included approximately 94% of the study region’s population, suggesting that most residents sought medical care at least once during the study period under Japan’s universal health insurance system.

Despite these limitations, this study used a large-scale database with 1-year postdisaster follow-up and employed multivariable and DID analyses, supporting the validity of our findings.

This large-scale observational study found that individuals affected by the 2018 Japan floods had higher rates of new prescriptions for asthma controller medications and exacerbation-related treatments than those not affected. These findings suggest a potential association between flood disasters and increased disease burden and health care utilization for adult asthma. Given the global increase in flood disasters because of climate change, our findings suggest the potential need for timely asthma care and the importance of maintaining health care access for affected populations.

## Disclosure statement

This research did not receive any specific grant from funding agencies in the public, commercial, or not-for-profit sectors.

Disclosure of potential conflict of interest: The authors declare that they have no relevant conflicts of interest.

## Supplementary data

Supplementary Tables S1-S5

Supplementary Figue S1
