# Supplementary material for: Impact of the 2018 Japan floods on asthma prescriptions: A longitudinal analysis
Source: J Allergy Clin Immunol Glob. 2026 Oct 5;6(1):100792. doi: 10.1016/j.jacig.2026.100792 (PMC13763423; doi:10.1016/j.jacig.2026.100792)
Supplement: Supplementary file 1 [file mmc1.docx]

**Supplementary Table 4. Characteristics of patients newly prescribed asthma controller medications after the disaster**
